# Dissecting the Role of a Basic Helix-Loop-Helix Transcription Factor, *SlbHLH22*, Under Salt and Drought Stresses in Transgenic *Solanum lycopersicum* L.

**DOI:** 10.3389/fpls.2019.00734

**Published:** 2019-06-04

**Authors:** Muhammad Waseem, Xiangyi Rong, Zhengguo Li

**Affiliations:** Key Laboratory of Plant Hormones and Development Regulation of Chongqing, School of Life Sciences, Chongqing University, Chongqing, China

**Keywords:** tomato, ROS scavenging system, proline, flavonoids, tolerance, drought, salinity

## Abstract

Environmental stresses, such as temperature, heavy metals, drought, cold, and microbial infections adversely damage various aspects of plant growth and development. Salinity and drought are among major hazardous factors, which adversity affects plant growth and productivity. Transcription factors, such as basic helix-loop-helix play critical roles in regulating plant physiological processes under abiotic stresses. In this study, we presented the characterization of a tomato *SlbHLH22* gene under abiotic stresses such as drought and salinity. Plants overexpressing *SlbHLH22* showed short height with small leaves and enhanced flavonoid accumulation. In wild type (WT) plant, the elevated levels of *SlbHLH22* were detected under salt and D-mannitol stresses. Subcellular localization analysis revealed that *SlbHLH22* protein was targeted to the nucleus in onion epidermal cells. Transactivation assay in yeast demonstrated that *SlbHLH22* had transcriptional activation ability. The transgenic plants overexpressing *SlbHLH22* displayed enhanced vigor and more tolerant to drought and salinity than WT. Overexpression of *SlbHLH22* significantly peaked the activities of catalase (CAT), superoxide dismutase (SOD), and peroxidase (POD) to minimize the impacts of reactive oxygen species such as H_2_O_2_, which was reduced significantly in transgenic plants along with Malondialdehyde (MDA). Moreover, the expression levels of ROS defense genes (*SlPOD, SlCAT, SlSOD*), ABA biosynthesis genes, proline biosynthesis, and flavonoids synthesis genes were also activated under salinity and drought. Taken together, our study implies that the overexpression of *SlbHLH22* improved tomato plant stress resistance by improving ROS scavenging system, increasing osmotic potential and enhanced accumulation of secondary metabolites in tomato plants.

## Introduction

Environmental stresses, such as salinity, drought, temperature, and pathogen invasion can adversely affect plant growth, development, and subsequently impacting its productivity. Plants have evolved various strategies to cope with such stresses, including biochemical, physiological, cellular, and molecular strategies (Thomashow, 1999; Nakashima et al., 2009). In general, plant responses to abiotic stresses are under the transcriptional control of various stress-induced genes and their activation or suppression results in a response to stimuli (Pang et al., 2011; Han et al., 2014; Sun et al., 2015).

The basic helix-loop-helix (bHLH) is one of the largest gene family in plants and has DNA binding and dimerization capabilities as bHLH domain existed. bHLH domain contains approximately 60 amino acid with a basic region and 2 functionally distinct regions of the HLH region in their protein sequences (Peng et al., 2013). bHLH proteins identified in various plant species, such as *Arabidopsis*, poplar, rice (Carretero-Paulet et al., 2010), maize (Zhang T. et al., 2018), grapes (Wang et al., 2018), and peaches (Zhang C. et al., 2018). However, this large number of bHLH genes in plants may leads to demonstrations that these plant bHLH proteins may severe as a key regulatory components in transcriptional activation or suppression of wide range of plant development, metabolic processes, and responses related to abiotic stresses (Peng et al., 2013).

The members of bHLH gene family have been reported to be related to responses to abiotic stresses, such as salinity, drought, and cold. *RsICE1* from *Raphanus sativus*, a stress-responsive bHLH TF enhances cold tolerance in rice through interacting with *CBF/DREB1* (Man et al., 2017). *ICE1* of *Pyrus ussuriensis* play pivotal roles in improving cold tolerance by increasing the transcriptional regulation of *PuDREB* via interaction with *PubHLH1* (Huang et al., 2015). Rice *OsbHLH148* could functions in JA-mediated drought tolerance (Seo et al., 2011), while *Arabidopsis bHLH122* acts as a transcriptional activator provides drought and osmotic resistance through enhanced proline accumulation and by activating ROS scavenging system (Liu et al., 2014, 2015). *Vitis vinifera VvbHLH1* has potential to improve tolerance to drought and salinity by regulating the accumulation of flavonoids and acts as regulator of ABA signaling (Wang et al., 2016). In addition to these diverse biological functions, the bHLH proteins were also reported to involve in various biosynthetic pathways such as anthocyanins and flavanols (Winkel-Shirley, 2001; Baudry et al., 2004).

*Solanum lycopersicum* L. is an ideal model plant for fruit development and its productivity is adversely affected by various abiotic stresses, such as salinity, drought, and temperature. The establishment of stress tolerant crop is key challenge in genetic engineering and biotechnology. We have previously shown that overexpression of *SlbHLH22* promotes flowering and fruit ripening and enhanced sensitivity to phytohormones with decreased fruit shelf life in tomato (Waseem and Li, 2019; Waseem et al., 2019). To investigate, whether overexpression of *SlbHLH22* improves plants tolerance to salinity and drought. The transgenic plants subjected to salinity and drought stresses. In this study, we found that *SlbHLH22* was peaked significantly under salt stress. Our studies demonstrated that the overexpression of *SlbHLH22* confers abiotic stress tolerance by regulating the expression of stress-inducible genes that are involved in physiological changes, including reactive oxygen species (ROS) scavenging system, abscisic acid (ABA) signaling, flavonoid biosynthesis pathway, and proline biosynthesis. We believe, our study might provide a new insight into the functional characterization of the bHLH gene family members during stress tolerance in tomato and other pant species.

## Materials and Methods

### Plant Growth Conditions and Collection

The surface sterilized seeds of *Solanum lycopersicum* L. cv. Micro-Tom wildtype (WT) and transgenic lines and empty vector (VC) were grown in green house under following conditions: 16 h/8 h light/dark cycle, 25°C/18°C day/night temperature, light intensity 250 μ mol m^−2^s^−1^, and 80% relative humidity. For gene expression analysis, plant parts, such as root, leaves, stem, flowers (in bud and fully opened), and flower parts (sepal, petal, carpel, and stamens) were harvested from 4-week-old plants (Waseem et al., 2018). For each sample, each plant part was collected from 10 plants were mixed and frozen in liquid nitrogen.

### Plasmid Construction, Transformation, and Generation of Transgenic Plants

For overexpression, the K303 expression vector (Gateway technology) under CaMV 35S promoter was constructed as described in Waseem et al. (2019). For RNAi a 217 bp long *SlbHLH22* fragment for sense and antisense silencing in pCAMBIA 2301 vector. The specific primers used for RNAi are listed in Supplementary Table S1. The transgenic line plants were generated by agrobacterium-mediated (*Agrobacterium tumefaciens* strain, GV310) transformation in WT tomato plants as described by Xian et al. (2014). The transgenic plants were screened on MS media supplemented with kanamycin (100 mg L^−1^). The generated kanamycin resistant seedlings were transferred to green house for further growth under control conditions and then verified with successful qRT-PCR. The homozygous T_3_ lines were used for further analysis.

### Subcellular Localization and Transcriptional Activity of *SlbHLH22*

The *SlbHLH22* ORF without the stop codon was amplified and cloned into the pGreen0029 vector. The recombinant plasmid containing the *SlbHLH22*-GFP fusion gene and the control plasmid with GFP alone were transformed into onion epidermal cells using *Agrobacterium*-mediated transformation as described by Sun et al. (2007). For *trans*-activation assays, to produce pBD- *SlbHLH22* the coding sequence of *SlbHLH22* was amplified and ligated into the yeast expression vector pGBKT7 (Clontech, United States). pBD-*SlbHLH22*, pGBKT7 (plasmid for negative control), and pGBKT7-53+pGADT7-T (plasmid combination for positive control) were transformed separately into the yeast strain AH109 according to the manufacturer’s protocol. Transformants were selected on SD/-Trp or SD/-Ade/-His/-Trp drop-out medium (Clontech, United States). After colony formation, the *trans*-activation activity of each protein was examined by comparing growth on permissive and selective medium and the activity of X-gal (40 μg mL^−1^, 5-bromo-4-chloro-3-indoxyl-α-D-galactopyranoside).

### Determination of Total Flavonoids

Total flavonoid contents were determined by the AlCl_3_ method as described by Koolen et al. (2013) with slight modifications. 2.5 g of tomato leaves (WT, 35S:*SlbHLH22* and RNAi lines) were ground in liquid nitrogen and dissolved in 70% (by vol.) ethanol solution and incubated at room temperature for 24 h. 1 mL of ethanol extract was diluted with 1 mL of AlCl_3_ (5%, w/v) and incubated for 1 h at room temperature. The mixture was centrifuged at 10,000 rpm for 10 min and supernatant was collected in a new tube. One volume of chloroform was added to remove chlorophylls. The mixture was centrifuged at 8000 rpm for 5 min and supernatant was used to measure absorbance at 430 nm. Total flavonoids were expressed in mg quercetin equivalent/g dry weight (Koolen et al., 2013).

### Salt and Drought Stress Assay

For salt, osmotic, and oxidative stresses, the leaves from 5-week-old WT seedling were sprayed with 200 mM NaCl (Waseem and Ahmad, 2019), 100 mM mannitol, and 100 mM hydrogen peroxide, respectively. Leaves were harvested at 0, 3, 6, 9, 12, and 24 h. Leaves harvested at 0 h were used as control. For each sample, leaves were collected from 10 plants, mixed and all the experiments were performed in triplicate.

For salinity and drought stresses, each 15 plants of WT and transgenic lines were placed in big pot, watered twice in week to make sure water was uniform in all pots and were grown under same light and temperature conditions. For salinity stress, 6-week-old plants were irrigated with 200 mM (200 mL per pot, 9 cm) NaCl for every 48 h in the following 18 days. However, for drought stress, withhold water for up to 30 days followed by rehydrated for 10 days. The control plants were watered normally. During treatment, the relative water content (RWC, %) and total chlorophyll content was assessed (Pan et al., 2012). The leaves at same development stage were harvested and store immediately at −80°C till further analysis.

### Assessment of Antioxidant Enzyme Activity and ABA Content Measurement

During salinity and drought treatment, leaves at same development stages were harvested from plants for antioxidant enzyme activity such as catalase (CAT. EC 1.11.1.6) superoxide dismutase (SOD, EC 1.15.1.1), peroxidase (POD, EC 1.11.1.7), H_2_O_2_ content, Malondialdehyde (MDA), soluble sugar content, and proline content assessment. MDA following method by Heath and Packer (1968). Briefly, about 0.5 g of tomato leaves were ground in 2 mL of the chilled reagent [0.25% (w/v) thio-barbituric acid in 10% (w/v) trichloroacetic acid]. The extracts were incubated at 100°C for 30 min, cooled to room temperature. The extracts were centrifuged at 12,000 rpm for 15 min and absorbance of the supernatant was measured at 450, 532, and 600 nm. The MDA content was calculated based on the following equation: 6.45 × (OD_532_–OD_600_) −0.559 × OD_450_.

Soluble sugar content was measured according to method described by Fukao et al. (2006). Proline contents were determined following Bates et al. (1973). About 0.5 g of tomato leaves were ground into powder with liquid nitrogen and extracted in 3% sulfosalicylic acid. After centrifuging at 12,000 rpm for 10 min, the supernatant (2 mL) was mixed with equal volume of ninhydrin reagent [2.5% (w/v) ninhydrin, 60% (v/v) glacial acetic acid, and 40% 6 M phosphoric acid] and of glacial acetic acid, incubated at 100°C for 40 min. The reaction was terminated in an ice bath. Then, the reaction mixture was extracted with 4 mL of toluene. The absorbance was measured at 520 nm with a UV-5200 spectrophotometer.

For SOD activity, 1 g of frozen leaves tomato leaves were homogenized in 5 ml of cold 20 mM HEPES buffer (pH 7.2, 1 mM EGTA, 210 mM mannitol, 70 mM sucrose) then centrifuged at 2,500 rpm for 5 min at 4°C. The enzyme activity SOD were measured following Mittova et al. (2000). Total protein from tomato leaves was extracted with 0.05 M potassium phosphate buffer (pH 7.0). After centrifuging at 12,000 rpm for 15 min at 4°C, the supernatant was used for the measurement of POD and CAT activities. POD activity was determined using the previously described method by Morohashi (2002) and Morohashi et al. (2003). The 5 mL reaction mixture contained 0.1 mL of the supernatant, 1 mL of 0.5% (v/v) H_2_O_2_, 2.9 mL of 0.05 M potassium phosphate buffer (pH 5.5), and 1 mL of 0.05 M guaiacol as substrates. The oxidation of guaiacol was monitored by the absorbance measured at 470 nm every 10 s. CAT activity was confirmed using a Catalase Assay Kit (Jiancheng Bioengineering Company, Nanjing, China) according to the manufacturer’s instructions. H_2_O_2_ content was determined according to instruction available in commercial kit from Jiancheng Bioengineering Company (Nanjing, China). For ABA quantification, ABA extracted from 1 g of leaves (WT, transgenic lines under stress, and mock) as described by Jia et al. (2011).

### RNA Extraction, cDNA Preparation, and qRT-PCR Analysis

Total RNA was extracted from all harvested samples using Invitrogen^TM^ TRIzol^®^ reagent (Thermo Fisher Scientific, New York, NY, United States) according to the manufacturer’s instruction. The RNA concentration was determined using NanoDrop Lite UV-Vis spectrophotometer (Thermo Fisher Scientific^TM^). The cDNA was synthesized with 2 μg of total RNA using PrimeScriptTM RT reagent Kit with gDNA Eraser (TaKaRA, Japan). All the primers (Supplementary Table S1) used in this study were designed in primer premier 5 (PREMIER Biosoft International, Palo Alto CA, United States). The real-time PCR was performed using SYBR^®^ Premix Ex Taq^TM^ II (TliRNaseH Plus) (Clontech, TaKaRa, Shiga, Japan) in 96 well plate, Bio-Rad CFX system (Bio-Rad, United States). The relative changes in gene expression was calculated by adopting 2^−Δ(ΔCT)^ method (Livak and Schmittgen, 2001) using *SlUBI3* (Solyc01g056940) as an internal control. All the experiments were performed in triplicate.

### Statistical Analysis

All the experiments were performed in triplicate, reproducible and were presented as means ± standard error (SE). Statistical analysis of data was performed using Sigmaplot 12.1. (SYSTAT and MYSTAT Products, United States, and Canada) and two-tailed Student’s *t*-tests for salinity and drought comparison or Dunnett’s tests were used to compare between WT, empty vector plants, and each overexpression line to determine significant differences. The significance values of *p* ≤ 0.01^∗∗^/0.05^∗^ were considered.

## Results

### *SlbHLH22* Expression in Tomato WT Plants

**FIGURE 1:**
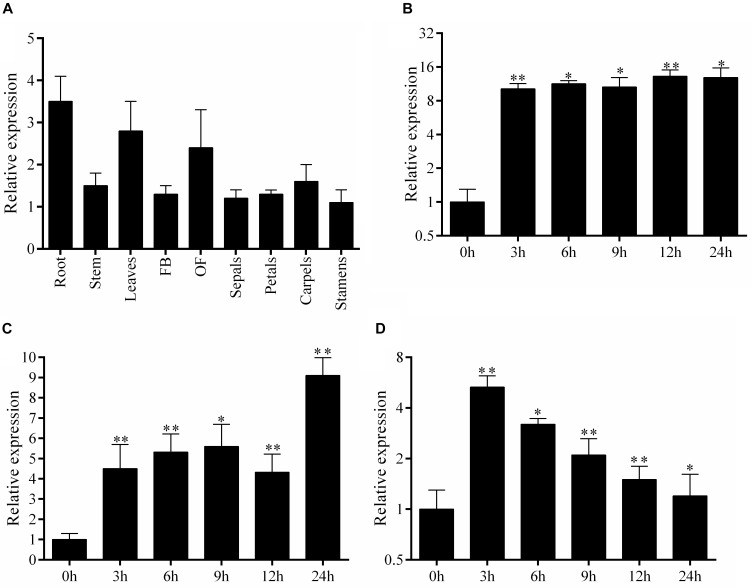
Endogenous expression profile of *SlbHLH22* and expression levels under abiotic stress. **(A)** Expression profile of *SlbHLH22* in different parts of WT plants, including root; stem; leaves; FB, flower bud; OF, opened flower; petals; sepals; stamens; and carpels. Expression analysis of *SlbHLH22* from leaves of WT spray with **(B)** 200 mM NaCl, **(C)** 100 mM D-mannitol, **(D)** 100 mM H_2_O_2_ over 0 to 24 h time intervals. Student’s *t*-tests were used in comparison. Data represent mean of ± SE for three independent biological replicates (*n* = 3). ^∗^ and ^∗∗^ represent the significant difference at *P*-value ≤ 0.01^∗∗^/0.05^∗^.

To gain insight into the roles of *SlbHLH22* in plant growth and development, we analyzed the expression profile of *SlbHLH22* in various plant parts using qRT-PCR. The results suggested that *SlbHLH22* expressed in all the tested tissues, but high expressions were found in leaves and flowers compared to root (Figure 1A). We further examined the expression of *SlbHLH22* response to osmotic and oxidative stresses. WT plants were treated with NaCl, H_2_O_2_, and D-mannitol. The results indicated that the transcript of *SlbHLH22* was upregulated after salt treatment across all time points (Figure 1B). For D-mannitol, *SlbHLH22* was upregulated after exposure, but was peaked at 24 h time point (Figure 1C). For H_2_O_2_ was only peaked at 3 h interval and was downregulated in the remaining time points (Figure 1D). The observation of expression profile for *SlbHLH22* indicated that *SlbHLH22* gene might be very important for plant resistance against abiotic stress.

### *SlbHLH22* Encodes a TF Targeted to the Nucleus

**FIGURE 2:**
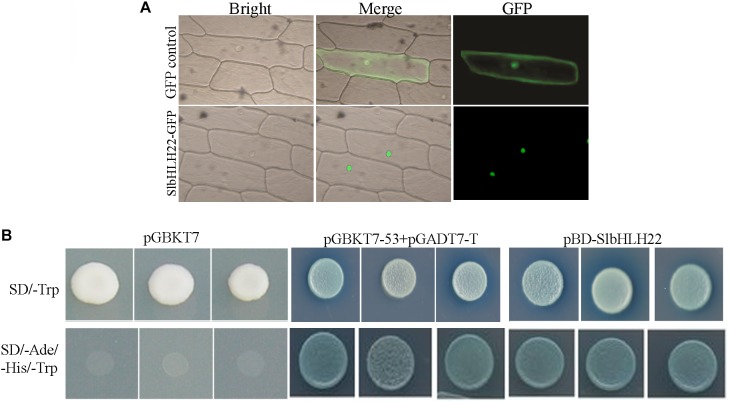
Subcellular localization and transcription activity of *SlbHLH22*. **(A)** The images were taken under bright light, merged, and in the dark field for the GFP-derived green fluorescence, respectively. **(B)** Analysis of the transactivation activity of *SlbHLH22*. Up, SD/-Trp-drop medium, below, SD/-Ade/-His/-Trp-drop medium both supplemented with X-gal for assaying yeast reporter (MEL1) gene.

To determine the subcellular localisation of *SlbHLH22* protein, the vector 35S-*SlbHLH22*-GFP was transiently expressed in living onion epidermal cells. Confocal imaging of protein fluorescence showed that the cells transformed with the vector containing GFP alone displayed fluorescence throughout the cells, whereas the green fluorescence signal of 35S-*SlbHLH22*-GFP was exclusively detected in the nucleus (Figure 2A). A Y2H experiment was used to examine the transcriptional activity of *SlbHLH22*. A GAL4 DNA-binding domain *SlbHLH22* fusion protein was expressed in yeast cells, which were then assayed for their ability to activate transcription from the GAL4 sequence. *SlbHLH22* promoted yeast growth in the absence of histidine and adenine, and showed X-α-gal activity, whereas the control vector pGBKT7 did not (Figure 2B). Moreover, string database was used to predict interaction network of *SlbHLH22* with other proteins. It was found that tomato *SlbHLH22* may interact with genes involved in flavonoid and ABA biosynthesis pathway include; CHS, CHI, PAL, PSY1, FLS, AAO, NCED, and ZEP (Supplementary Figure S1). Taken together, our results suggested that *SlbHLH22* has transcriptional activity and is targeted to the nucleus in plant cells.

### Phenotypic Characterization of Transgenic Tomato With *SlbHLH22*

**FIGURE 3:**
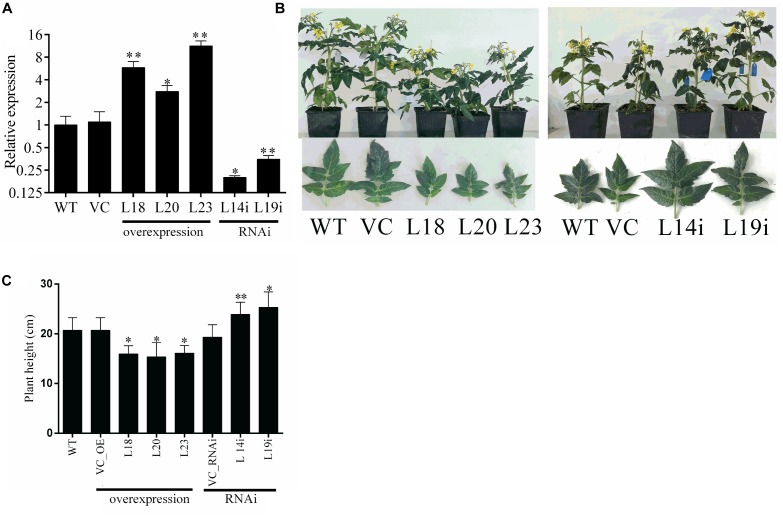
Transgenic line generation and phenotypic characterization of WT, empty vector, and transgenic plants. **(A)** Expression levels of *SlbHLH22* in WT, empty vector (VC), and transgenic lines (L18, L20, L23) overexpressing and RNAi-silencing (L14i and L19i) of *SlbHLH22* in leaves. Dunnett’s tests was used in the comparison between WT, VC, and *SlbHLH22* overexpression lines (L18, L20, L23) **(B)** 6-week-old WT, VC, and three independent transgenic lines (L18, L20, L23, L14i, and L19i) plants. Phenotype of fifth leaves in WT, empty vector (VC) plants, and in *SlbHLH22* transgenic line plants **(C)** Height of plants WT, VC, and transgenic lines. Data represent mean of ± SE for three independent biological replicates (*n* = 3) and each replicate contained 15 plants. ^∗^ and ^∗∗^ represent the significant difference as determined by *t*-test (*P*-value ≤ 0.01^∗∗^/0.05^∗^).

To further study the function of *SlbHLH22* gene, the transgenic plant lines were generated by overexpressing the ORF and RNAi silencing by agrobacterium-mediated transformation. Three independent transgenic lines for overexpression (L18, L20, L23) and two RNAi lines (L14i and L19i) were detected exhibiting significant changes in expression fold (Figure 3A). Two transgenic plant lines L18 and L23 for overexpression and two lines for RNAi L14i and L19i were selected for further characterization. The transgenic plants showed pleiotropic phenotypes, such as plant height and leaf size (Figure 3B,C). Our results displayed that the tomato *SlbHLH22* have remarkable effects on development of tomato plant.

### *SlbHLH22* Enhances Transgenic Tomato Plant Resistance to Salt and Drought

**FIGURE 4:**
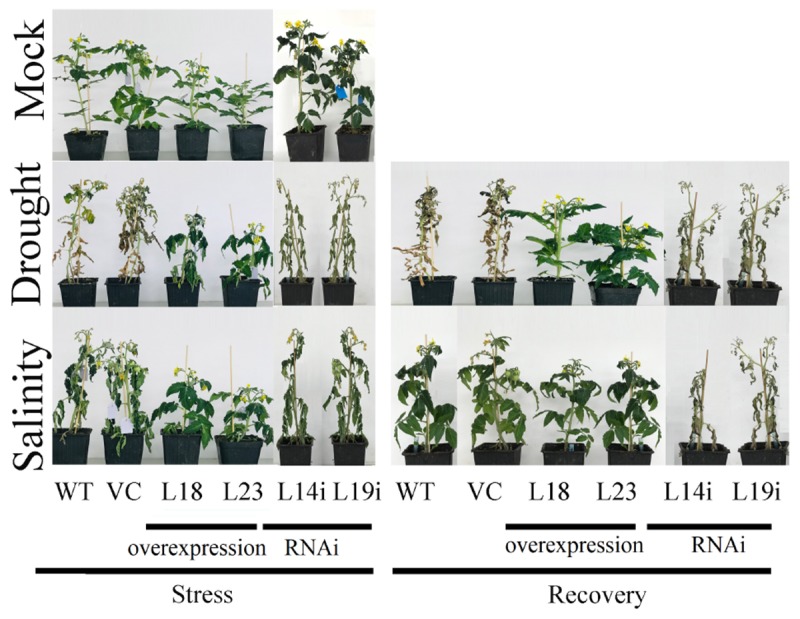
Drought and salinity assay. Phenotypes obtained after 30 days drought and 18 days salinity stress and recovery from drought and salinity for 10 days.

As it was observed, *SlbHLH22* expressions was activated by salinity. Thus, we hypothesized that *SlbHLH22* might increase the resistance of transgenic plants to salinity and drought. To prove it, we explore the role the performance of transgenic and WT plants treated with salt solution for 18 days and deprived of water for 30 days. As shown in Figure 4, significant morphological changes were observed between transgenic plant lines with *SlbHLH22* and WT plants after stresses. However, after stresses, the overexpression plant lines showed slight changes in their physical appearance, but RNAi lines and WT plants showed typical severe desiccation symptoms (Figure 4). In comparison, WT and RANi lines plants under drought showed severe damages than under salt treatment. However, upon exposure to normal conditions for 10 days, the overexpression plants recover very fast, but RNAi and WT plants under drought stress was unable to recover (Figure 4). The relative water content (RWC %) and total chlorophyll content was decreased in WT and transgenic lines during treatment, but relatively higher in the overexpression plant lines (Supplementary Figures S2A,B).

### Upregulation of Flavonoid Accumulation Under Salt and Drought Stress

The total flavonoid contents were assessed in WT and transgenic lines under normal conditions and under salinity and drought stresses. It was found that the total flavonoid contents in overexpression plant lines were induced and suppressed significantly in RNAi lines compared to WT (Supplementary Figure S3). The expression levels of genes in the flavonoid biosynthesis pathway were further analyzed at molecular level in the overexpression and RNAi lines. The results indicated that the transcript levels of the flavonoid biosynthesis genes, such as *SlCHS, SlCHI, SlF3’H, SlF3H, SlFLS*, and *SlPAL* were peaked in overexpression lines than in WT. In *SlbHLH22*-RNAi lines the expression of all genes significantly downregulated than in WT plants (Supplementary Figure S3). These results demonstrating that *SlbHLH22* affect flavonoid accumulation by modulating flavonoid biosynthesis pathway.

### Improved Antioxidant Activity in Transgenic Plants

**FIGURE 5:**
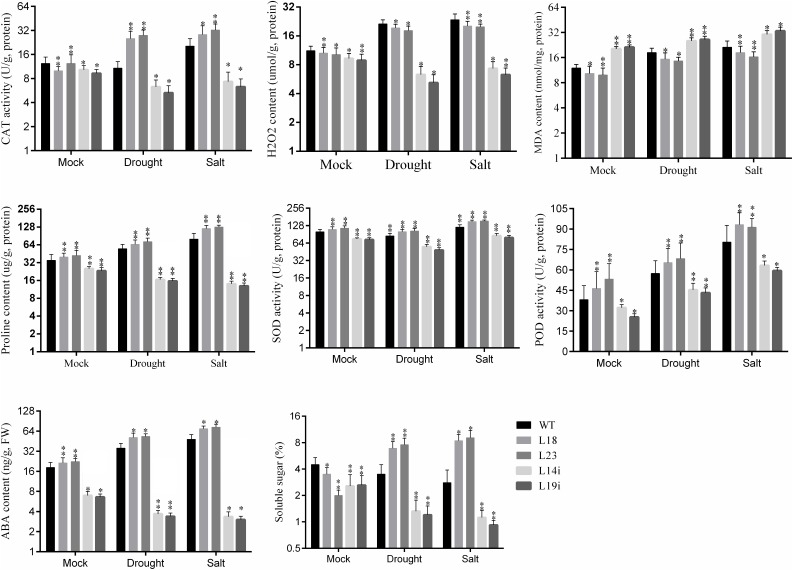
ABA content, soluble sugar, Malondialdehyde (MDA), hydrogen peroxide (H_2_O_2_) and antioxidant enzymes [catalase (CAT), superoxide dismutase (SOD), and peroxidase (POD)] activities in WT and transgenic lines (L18 and L23) under drought and salinity. Data represent mean of ± SE for three independent biological replicates (*n* = 3) and each replicate contained 6 plants. ^∗^ and ^∗∗^ represent the significant difference as determined by *t*-test (*P*-value ≤ 0.01^∗∗^/0.05^∗^).

Salinity and drought lead to production of reactive oxygen species (ROS), that cause damages to membrane structure (Zhai et al., 2016). We investigated the changes in the accumulation of H_2_O_2_ in transgenic lines and WT plant under salinity and drought stresses. It was found that more H_2_O_2_ accumulated in WT plant than in transgenic lines (Figure 5). To explore the possible physiological mechanism responsible for the increased drought and salt tolerance, we compared the changes in contents of proline, MDA concentration, and total soluble sugars in the leaves from transgenic and WT plants grown under normal and stress conditions. MDA content was significantly peaked in RNAi-lines and WT plants under drought and salt stresses compared to overexpression lines (Figure 5). It was examined that the soluble sugars were accumulated more in overexpression lines than in RNAi-lines and WT plant, after salt and drought stresses (Figure 5). Furthermore, we examined the enzyme activities of antioxidants enzymes such as SOD, POD, and CAT of the leaves from transgenic plant lines and WT plant under stresses and normal growth. The enzymatic activities of SOD, POD, and CAT in transgenic lines and WT were almost same under normal conditions. For drought stress, the activities of CAT and SOD were significantly upregulated in overexpression lines as compared with WT and *SlbHLH22*-RNAi lines. However, POD activity was 10 points more in *SlbHLH22* overexpression lines than in the WT and RNAi (Figure 5). The activities of SOD, POD, and CAT were significantly upregulated in overexpression lines under salt stress in compassion with the mocked corresponding transgenic lines, RNAi lines, and WT plants. The proline contents were increased in overexpression plant lines under drought and salt stress in mocked transgenic lines but peaked in transgenic lines under salt stress. However, in RNAi lines proline content was significantly downregulated than WT plants. It was found that the overexpression plant accumulated more prolines contents under salinity stress than under drought (Figure 5). Collectively, our results show that *SlbHLH22* in tomato can help improve the resistance of transgenic plant to salinity and drought stresses.

### Up-Regulation of ABA and Stress Related Pathways in Tomato

**FIGURE 6:**
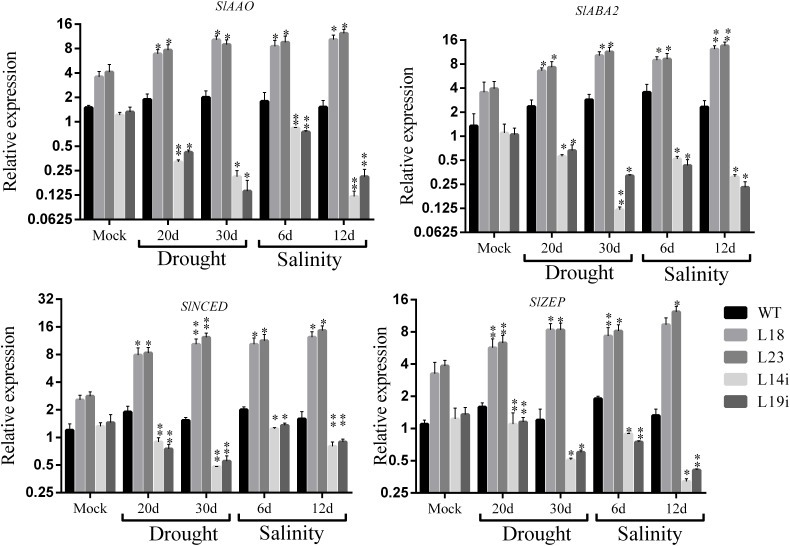
Expression profile of ABA biosynthesis related genes in WT and transgenic lines (L18 and L23) under drought and salinity. Data represent mean of ± SE for three independent biological replicates (*n* = 3). ^∗^ and ^∗∗^ represent the significant difference as determined by *t*-test (*P*-value ≤ 0.01^∗∗^/0.05^∗^).

The overexpression of *SlbHLH22* improved plant lines resistance against salinity and drought leads us to examine whether the overexpression of *SlbHLH22* affects the endogenous level of ABA and its biosynthesis genes in transgenic tomato. We measured the ABA levels in leaves of transgenic lines overexpressing *SlbHLH22* and WT plants under normal growth conditions and under stresses. The results showed a significant difference in ABA levels in WT and transgenic lines under normal conditions. However, the endogenous ABA contents was significantly higher in transgenic lines than that in the WT under salt and drought stresses (Figure 5). To further insight into the role of *SlbHLH22* in ABA biosynthesis, we examined ABA levels by generating RNAi lines. It was observed that ABA level in RNAi is downregulated than WT under mock and salinity and drought stress (Figure 5). To ascertain the molecular mechanism involved in ABA biosynthesis, we analyzed the expression level of ABA biosynthesis genes between transgenic lines and WT plants. The results indicated that the expression of genes involved in ABA biosynthesis, such as *SlAAO, SlABA2, SlNCED*, and *SlZEP* were upregulated in transgenic lines under salt and drought than in WT and RNAi lines (Figure 6). Meanwhile, we investigated the expression profiles of genes involved in ROS scavenging system, including *SlCAT, SlPOD, SlSOD* (Supplementary Figure S4), and proline biosynthesis such as *SlP5CS* and *SlP5CR* (Supplementary Figure S4) in WT and transgenic lines under normal and stress conditions. The results suggested minor changes in the expression levels of *SlCAT, SlPOD*, and *SlSOD* in WT plants under normal and stress conditions but significantly downregulated in *SlbHLH22*-RNAi lines. Moreover, the transcripts of *SlCAT, SlPOD*, and *SlSOD* accumulated more in overexpression lines under stresses than WT and mocked corresponding overexpression plant lines grown under normal conditions (Supplementary Figure S4). *SlP5CS* and *SlP5CR* are two key genes in proline biosynthesis. The expression levels of *SlP5CS* and *SlP5CR* were upregulated under stress in WT plants, but more high transcript levels were detected in overexpressing plant lines grown under stress treatments (Supplementary Figure S4). These observations suggested that tomato *SlbHLH22* may enhance tomato resistance to salt and drought stresses through ABA and/or other pathways.

## Discussion

Plants, being sessile organisms encounter a range of biotic and abiotic stresses, such as pathogen invasion, temperature, light, drought, salinity, and so on. These stresses negatively affect growth, yield, and survival rate of plants (Sharma et al., 2013). Plants develop various mechanisms to counter the effects of these stresses, which enables them to adopt under such conditions (Gerszberg and Hnatuszko-Konka, 2017). Molecular biology and biotechnology approaches enables us to find out large number of genes, including transcription factors (TFs) that play critical roles in spatial and temporal genes expression during stress resistance which could be induced by one or more abiotic and biotic stresses (Ashrafi-Dehkordi et al., 2018). Moreover, the functions of most TFs are still unknown. The current study is important in elucidating the roles of a basic helix-loop-helix transcription factor gene, *SlbHLH22*, in tomato in response to salinity and drought stresses.

The results of subcellular localization of *SlbHLH22*-GFP show that the GFP signal was in the nucleus (Figure 2A), which suggests that *SlbHLH22* might function in nucleus. Transcriptional activation assay suggested that *SlbHLH22* showed transcriptional activation ability (Figure 2B). Further investigation should be performed to examine whether *SlbHLH22* protein can function as activators or repressors of transcription in plants. In this study, phenotype analysis showed that the transgenic plant lines displayed changes in plant height and in leaves (Figure 3B) and altered flavonoid accumulation (Supplementary Figure S3). The plants overexpressing *SlbHLH22* showed delay symptoms of necrosis, wilting, and leaf senescence than in *SlbHLH22*-RNAi and WT plants grown under salinity and drought stresses (Figure 4). The total chlorophyll and relative water content in leaves remain high in overexpressing lines (Supplementary Figures S2A,B). These findings suggested that the overexpression of *SlbHLH22* enhances the tomato plant tolerance to salinity and drought. Flavonoids are a diverse group of naturally occurring secondary metabolites in plants, have strong antioxidant capacity (Verhoeyen et al., 2002). Flavonoids can enhance plant tolerance to drought and salinity stresses due to their ability to remove superoxide, peroxides, and free radicals produced during stress (Balasundram et al., 2006; Gao et al., 2011). For an instance, in *Vitis vinifera*, the overexpression of *VvbHLH01* in *Arabidopsis* increased resistance to drought and salinity through improves flavonoids accumulation (Wang et al., 2016). The flavonoid biosynthesis genes such as *CHS, F3H, FDR* were also upregulated in transgenic *Arabidopsis* plants overexpressing *AtbHLH8* (Shin et al., 2007). Thus, we hypothesized that the accumulation of flavonoids can enhance tolerance to osmotic and oxidative stresses. In order to validate it, the tomato transgenic plant lines were subjected to these stresses. It was observed that plants overexpressing *SlbHLH22* accumulate more flavonoids and the expression levels of flavonoid biosynthesis genes were upregulated in transgenic plant lines grown under salinity and drought stress than in WT and silencing lines plant (Supplementary Figure S3). This implying that higher the flavonoid accumulation more will be the tolerance to the oxidative stresses.

**FIGURE 7:**
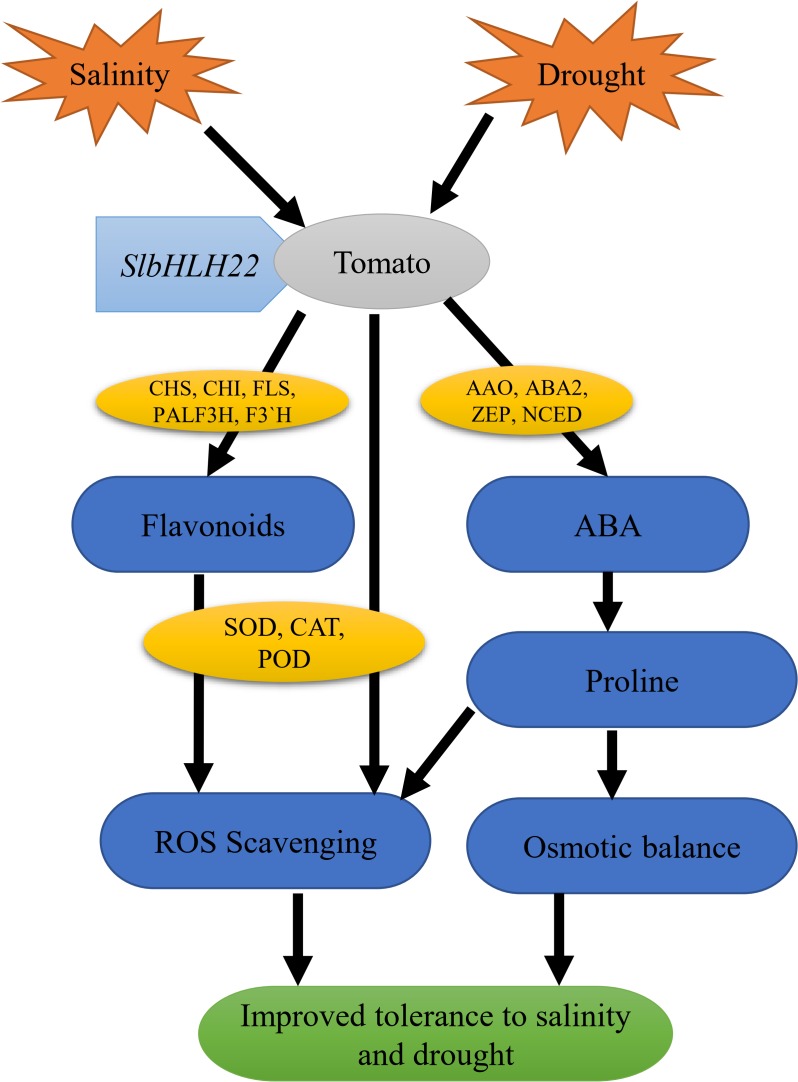
Hypothetical model of *SlbHLH22* gene involved in improving drought and salinity tolerance in tomato. Overexpression of *SlbHLH22* induced the expression of genes involved in flavonoid biosynthesis, ABA biosynthesis pathway and ROS scavenging genes. This subsequently resulted in altered physiology such as increased ABA accumulation, proline content, and enhanced CAT, POD, SOD activities with reduced ROS accumulation. This led to improve tolerance under abiotic stress.

Abscisic acid is a prime mediator known to regulate various plant physiological processes in adaptive responses to abiotic and biotics stresses (Umezawa et al., 2010). ABA regulates the expression of various stress-induced genes involved in proline, carbohydrate, and LEA biosynthesis that help plant to maintain the cellular water content and protect cellular proteins or enzymes (Verslues et al., 2006; Sreenivasulu et al., 2012). In our study, the ABA significantly peak under saline and drought stresses (Figure 6) and genes involved in ABA biosynthesis, such as *SlAAO, SlABA2, SlZEP*, and *SlNCED* were also upregulated (Figure 6). Similarly, this net upregulation of ABA can promote the expression of proline biosynthesis genes such as *SlP5CS* and *SlP5CR*, the key genes in proline biosynthesis (Supplementary Figure S5) and encourage soluble sugar content accumulation (Figure 5). We supposed that the overexpression of *SlbHLH22* enhances the plant ability to scavenge reactive oxygen species (ROS). In plants, ROS scavenging enzymes such as POD, SOD, and CAT helps to minimize osmotic and oxidative damages to plasma membrane integrity, proteins, and cellular enzymes (Zhang et al., 2012; Zhai et al., 2016). It was found that H_2_O_2_ accumulated more in WT plants then the transgenic plants grown under salinity and drought, (Figure 5). This subsequently leads to systematic upregulation of ROS scavenging genes, such as *SlSOD, SlPOD*, and *SlCAT* in WT plants (Supplementary Figure S4). Moreover, the elevated levels of MDA damages the integrity of phospholipid bilayer membranes, which reduces plant tolerance to salt and drought (Zou et al., 2012). The MDA levels were decreased in tomato transgenic plant lines overexpressing *SlbHLH22* (Figure 5). Thus, our results supported the fact that the overexpression of *SlbHLH22* enhances tomato tolerance to drought and salinity due to elevated levels of ABA. The upregulation of ABA, proline biosynthesis genes, and genes involved in ROS scavenging system lead to enhance ability of transgenic tomato plants to cope with applied stress (Figure 7).

## Conclusion

In summary, the overexpression of tomato bHLH TF gene, *SlbHLH22*, enhances resistance to drought and salinity by increased in flavonoids accumulation, ABA accumulation and ABA-induced-stress related pathways. All these physiological changes lead to improve plant ability to survive under abiotic stress conditions. This study not only provides the evidences of bHLH roles in resisting abiotic stresses but, also helps to improve our understanding about their role in abiotic stresses.

## Data Availability

No datasets were generated or analyzed for this study.

## Author Contributions

MW designed and performed all the experiments and data analysis. XR performed the data analysis. MW drafted the manuscript. ZL revised the manuscript. All authors have read and approved the final manuscript.

## Conflict of Interest Statement

The authors declare that the research was conducted in the absence of any commercial or financial relationships that could be construed as a potential conflict of interest.

## Supplementary Material

FIGURE S1 *In silico* bioinformatics analysis of *SlbHLH22* protein interaction network predicted using STRING program.

FIGURE S2 Relative water and chlorophyll content of WT and transgenic line plants under salinity and drought stresses. Relative water content (%), total chlorophyll content during various time points under **(A)** salinity and **(B)** drought stresses, respectively. Data represent mean of ± SE for three independent biological replicates (*n* = 3) and each replicate contained 15 plants. ^∗^ and ^∗∗^ represent the significant difference as determined by *t*-test (*P*-value ≤ 0.01^∗∗^/0.05^∗^).

FIGURE S3 Flavonoid content and expression profile of flavonoid biosynthesis genes in WT and transgenic lines under drought and salinity. Data represent mean of ± SE for three independent biological replicates (*n* = 3). ^∗^ and ^∗∗^ represent the significant difference as determined by *t*-test (*P*-value ≤ 0.01^∗∗^/0.05^∗^). ^1^Expressed in mg quercetin equivalent/g dry weight.

FIGURE S4 Expression profile of ROS scavenging related genes catalase (*SlCAT*) peroxidase (*SlPOD*), superoxide dismutase (*SlSOD*) genes in WT and transgenic lines under drought and salinity. Data represent mean of ± SE for three independent biological replicates (*n* = 3). ^∗^ and ^∗∗^ represent the significant difference as determined by *t*-test (*P*-value ≤ 0.01^∗∗^/0.05^∗^).

FIGURE S5 Expression profile of proline biosynthesis genes in WT and transgenic lines under drought and salinity. Data represent mean of ± SE for three independent biological replicates (*n* = 3). ^∗^ and ^∗∗^ represent the significant difference as determined by *t*-test (*P*-value 0.01^∗∗^/0.05^∗^).

TABLE S1 List of primer used in cloning and qRT-PCR for the expression analysis in this study.

## Abbreviations

ABA: abscisic acid
bHLH: basic helix-loop-helix
CAT: catalase
CBF: C-repeat binding factor
DREB: dehydration responsive element-binding protein
H_2_O_2_: hydrogen peroxide
MDA: Malondialdehyde
POD: peroxide dismutase
ROS: reactive oxygen scavenging system
SOD: superoxide dismutase
TFs: transcription factors.
