# Supplementary figures and images for: Dissecting the Role of a Basic Helix-Loop-Helix Transcription Factor, SlbHLH22, Under Salt and Drought Stresses in Transgenic Solanum lycopersicum L
Source: Front Plant Sci. 2019 Jun 4;10:734. doi: 10.3389/fpls.2019.00734 (PMC6558761; doi:10.3389/fpls.2019.00734)

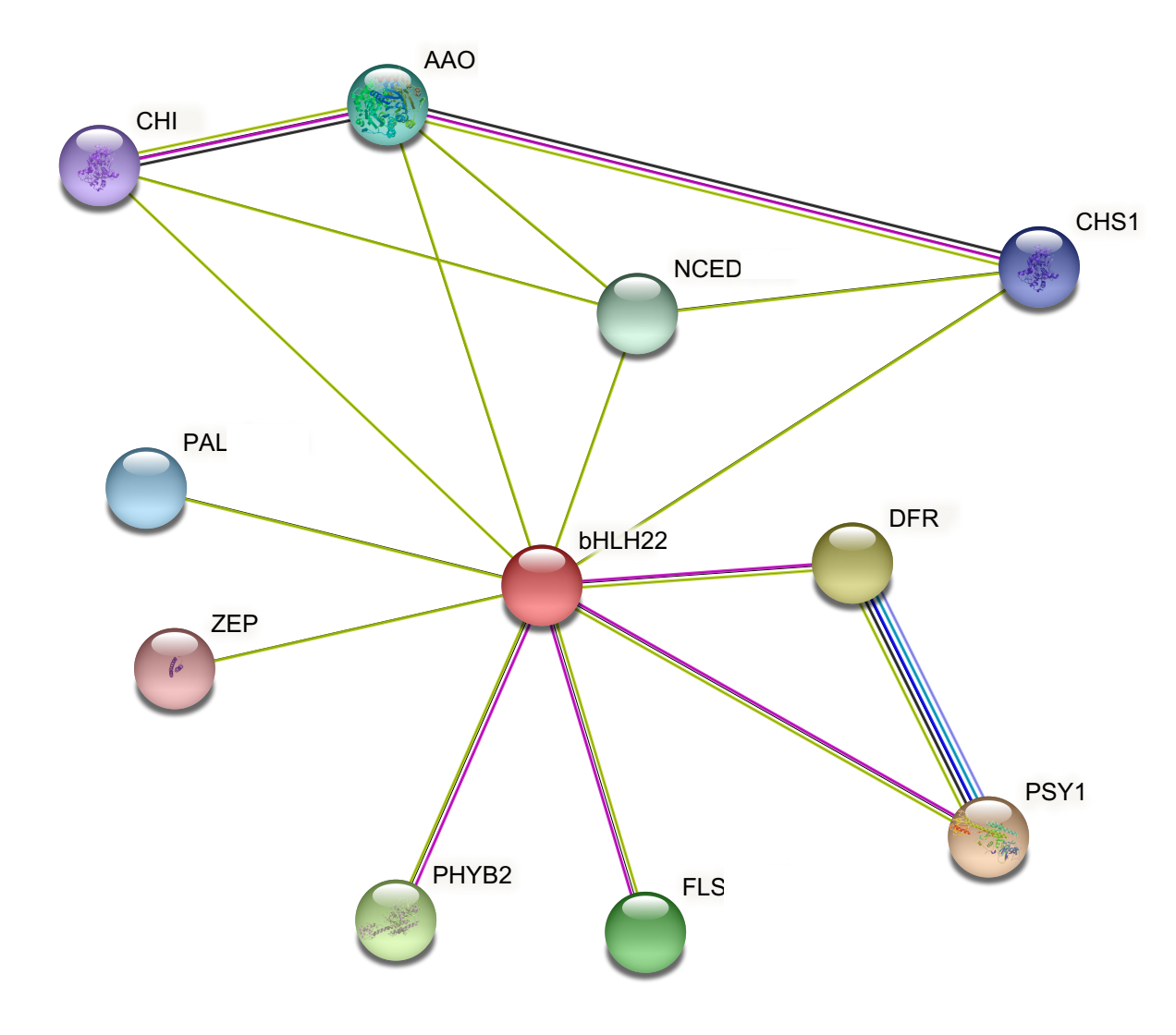

Supplement: FIGURE S1 — In silico bioinformatics analysis of SlbHLH22 protein interaction network predicted using STRING program. [file Image_1.TIF]

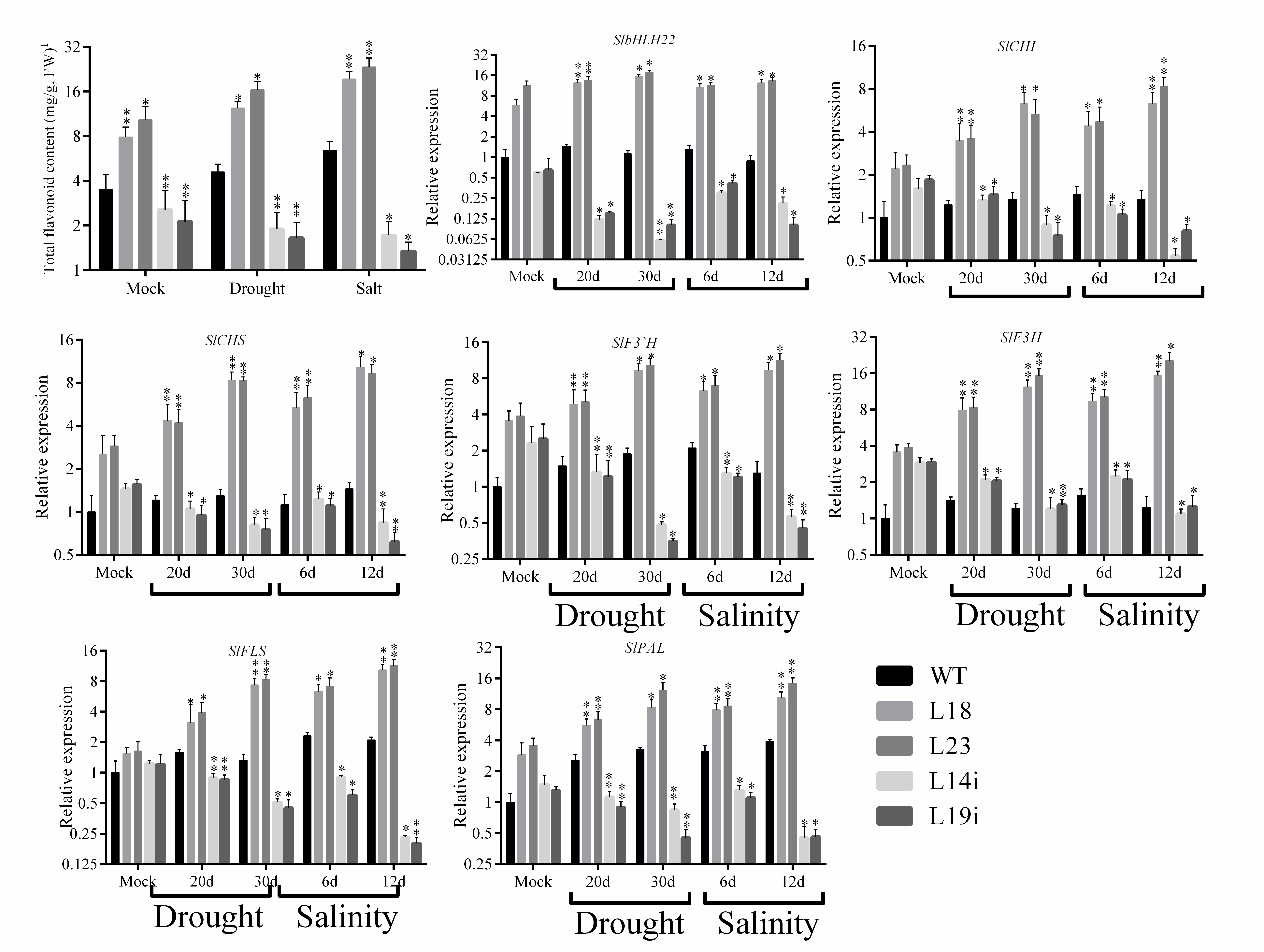

Supplement: FIGURE S3 — Flavonoid content and expression profile of flavonoid biosynthesis genes in WT and transgenic lines under drought and salinity. Data represent mean of ± SE for three independent biological replicates (n = 3). ∗ and ∗∗ represent the significant difference as determined by t-test (P-value ≤ 0.01∗∗/0.05∗). 1Expressed in mg quercetin equivalent/g dry weight. [file Image_3.TIF]

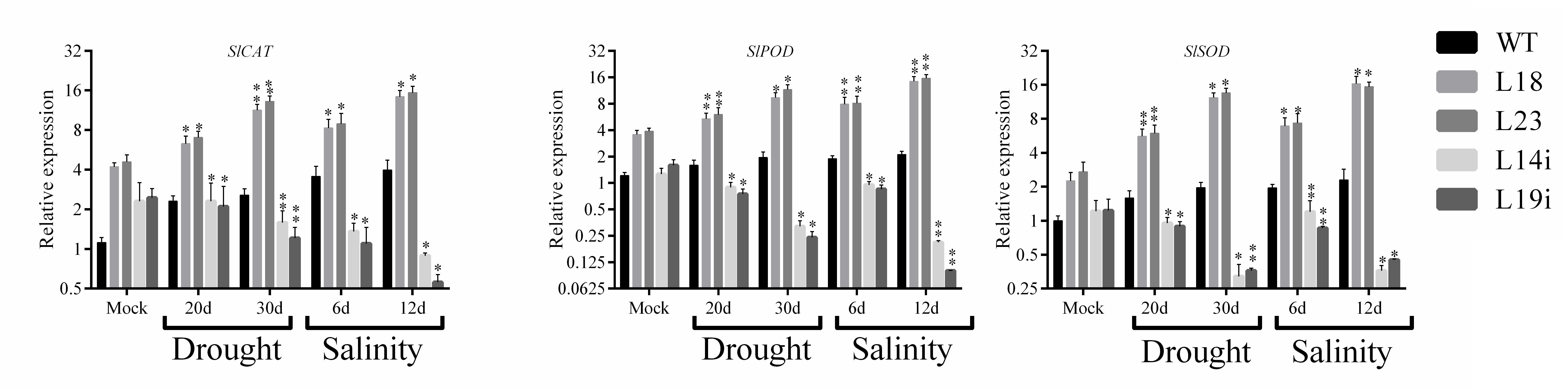

Supplement: FIGURE S4 — Expression profile of ROS scavenging related genes catalase (SlCAT) peroxidase (SlPOD), superoxide dismutase (SlSOD) genes in WT and transgenic lines under drought and salinity. Data represent mean of ± SE for three independent biological replicates (n = 3). ∗ and ∗∗ represent the significant difference as determined by t-test (P-value ≤ 0.01∗∗/0.05∗). [file Image_4.TIF]

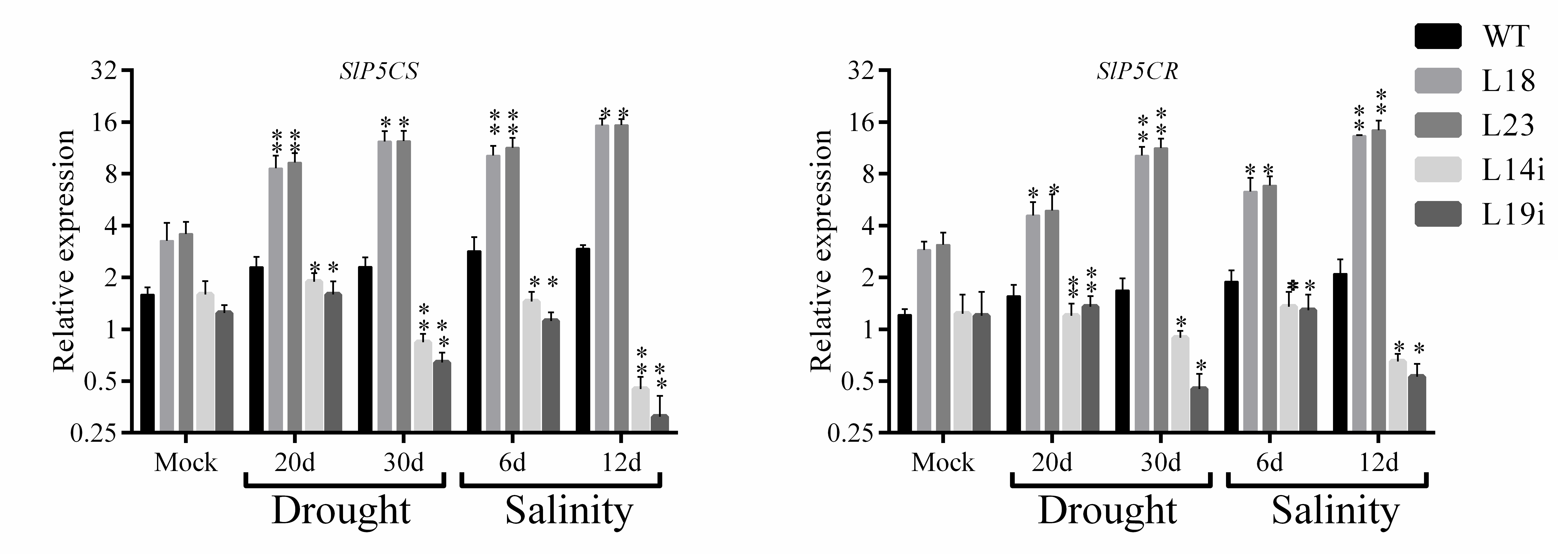

Supplement: FIGURE S5 — Expression profile of proline biosynthesis genes in WT and transgenic lines under drought and salinity. Data represent mean of ± SE for three independent biological replicates (n = 3). ∗ and ∗∗ represent the significant difference as determined by t-test (P-value 0.01∗∗/0.05∗). [file Image_5.TIF]
